# Rutin Mediated Apoptotic Cell Death in Caski Cervical Cancer Cells via *Notch*-*1* and *Hes*-*1* Downregulation

**DOI:** 10.3390/life11080761

**Published:** 2021-07-28

**Authors:** Fahad Khan, Pratibha Pandey, Niraj Kumar Jha, Mohammad Khalid, Shreesh Ojha

**Affiliations:** 1 Noida Institute of Engineering and Technology, Greater Noida 201306, India; fahadkhan.bio@niet.co.in; 2 Department of Biotechnology, School of Engineering & Technology (SET), Sharda University, Greater Noida 201310, India; 3 Department of Pharmacognosy, College of Pharmacy, Prince Sattam Bin Abdul Aziz University, P.O. Box 173, Al-Kharj 11942, Saudi Arabia; drkhalid8811@gmail.com; 4 Department of Pharmacology and Therapeutics, College of Medicine and Health Sciences, United Arab Emirates University, Al Ain P.O. Box 15551, United Arab Emirates; shreeshojha@uaeu.ac.ae

**Keywords:** cervical cancer, *Notch*-*1*, *Hes*-*1*, rutin, apoptosis, cell cycle arrest

## Abstract

Natural dietary molecules such as flavonoids have been recognized for their immense potential in cancer therapeutics with several health benefits. *Hes*-*1* and *Notch*-*1* overexpression has been associated with the progression of cervical cancer. However, the apoptosis-inducing potential of one such potent flavanol against these two key components of the Notch signaling pathway in cervical cancer has not been elucidated to date. Therefore, in this study, we performed several in vitro assays to gain detailed insight about the apoptotic inducing effect of rutin as well as its modulatory effect on *Notch*-*1* and *Hes*-*1* in cervical cancer cells. The results indicated that rutin led to a dose-dependent antiproliferative effects on Caski cervical cancer cells. DAPI and Mitotracker red staining revealed that rutin induced significant apoptotic effects via caspase-3/9 activation, ROS generation, and alteration in *Bax*/*Bcl2* mRNA expression. Cell cycle analysis resulted in the arrest of cell cycle progression in G0/G1 that was associated with a reduced expression of *CDK4* and *Cyclin D1*. The gene expression analysis further revealed that rutin treatment decreases *Notch*-*1* and *Hes*-*1* mRNA expression. Altogether, these results showed that rutin showed potent anticancer effects in human cervical cancer Caski cells by triggering apoptosis, G0/G1 phase arrest, and downregulating the level of *Notch*-*1* and *Hes*-*1* of the Notch signaling pathway.

## 1. Introduction

Cervical cancer is the uncontrolled proliferation of malignant cells originating within the uterine cervix. Cervical cancer is the second most common malignancy and the leading cause of mortality among women worldwide [1,2,3]. According to the 2018 reports, the new cases of cervical cancer in India were reported to be 96,922 and with a number of mortalities estimated to be 60,078, which indicates an alarming situation [4,5]. Despite advanced screening programs, therapeutic approaches, and vaccinations, the burden of mortality continues to rise. The reason behind the increased mortality rate could be the mounting drug resistance in cancer cells which leads to cancer relapse. Drug resistance usually arises due to the stimulation of proto-oncogene, deregulated cell signaling pathways, alteration in drug targets, or due to variations in the tumor microenvironment [6]. Thus, novel drug candidates with specific cellular targets and a concurrent impact on multiple signaling pathways need to be explored for the management of cervical cancer [7].

In recent decades, the molecular components of signaling pathways have gained wider recognition in cancer therapeutics. In the context of abnormal cell signaling with carcinogenesis, an aberrant Notch signaling cascade was associated with various types of tumors; however, there are few reports about the involvement of Notch signaling in cervical cancer. The Notch signaling pathway was considered an important regulator of cell proliferation, apoptosis, and tumorigenesis in humans [8,9,10]. Several studies have established the involvement of an aberrant Notch signaling cascade in different carcinomas [11]. The association of an increased expression of Notch receptors and ligand–protein interaction has been reported in the development and progression of cervical cancer [12]. Therefore, targeting such key components of the Notch signaling pathway could be a promising approach for finding a better management of cervical cancer.

In recent years, there has been great interest in exploring the chemopreventive potential of phytochemicals including flavonoids, polyphenolic compounds for the discovery of new drugs against cancers with minimal or no side effects [13,14,15]. One such flavonoid is rutin (rutoside or quercetin-3-O-rutinoside or sophorin), which is a glycoside comprised of the flavonol quercetin and rutinose, and commonly present in various plants, such as oranges, apples, grapes, tea, cherries, and grapefruit. Numerous biological and pharmacological properties of rutin were investigated, including anti-inflammatory, antioxidant, pro-apoptotic, antiangiogenic, and antitumorigenic activities [16,17,18,19]. 

In a recent study, we assessed the anticancer effects of rutin against the HPV negative C33A cell line by inducing cell cycle arrest and apoptosis [20]. Previous literature has recommended the utility of plant-based compounds in finding a promising and cost-effective approach via targeting mutated key molecules of signaling pathways that are involved in carcinogenesis [21,22,23]. Therefore, this study is directed towards exploring the inhibitory potential as well as the detailed underlying mechanism of rutin (bioflavonoid) via targeting two crucial components of the Notch signaling pathway in HPV 16 positive cervical cancer cells. 

## 2. Materials and Methods

### 2.1. Chemicals and Reagents

Rutin hydrate, 5-fluorouracil, propidium iodide (PI), DAPI (4′,6-diamidino-2-phenylindole), 2′,7′-dichlorodihydrofluorescein diacetate (DCFH-DA), MitoTracker Red CMX ROS, and Cytotoxicity Cell Death Kit were purchased from Sigma (St. Louis, MO, USA). Fetal bovine serum (FBS), 3-(4,5-dimethylthiazol-2-yl)-2,5-diphenyl tetrazolium bromide (MTT), and other chemicals were purchased from Himedia India, Ltd. (Mumbai, India). Verso cDNA synthesis kit and DyNAmoColorFlash SYBR Green qPCR Kit were purchased from Thermoscientific, USA. Caspase-3 and -9 assay kits were purchased from BioVision Milpitas, CA, USA. 

### 2.2. Cell Culture

An HPV16 positive human cervical cancer Caski cell line was obtained from the National Center for Cell Science, Pune (India). These cells were grown and maintained in RPMI (Roswell Park Memorial Institute)-1640 medium supplemented with 10% fetal bovine serum containing 1% antibiotic–antimycotic solution. Caski cells were cultured in a humidified chamber with 5% CO_2_ at 37 °C.

### 2.3. In Vitro Cell Viability Assay

MTT assay was performed to analyze the effect of rutin as well as 5-fluorouracil on Caski cells. To do so, approximately 5 × 10^3^ cervical cancer Caski cells per well were plated, in 96-well tissue culture plates and incubated for 24 h. After incubation, Caski cells were treated with increasing concentrations of rutin (60–180 µM). Additionally, cervical cancer Caski cells were also incubated with varying concentrations of 5-fluorouracil (5-FU) (1–5 µM) as a positive control for 24 h. After 24 h treatment with rutin and 5-FU, 20 µL MTT dye was added to each well and incubated for 3 h at 37 °C. Subsequently, 100 μL DMSO (dimethyl sulfoxide) was added to each well to solubilize the formazan and the absorbance was then recorded at 540 nm in an ELISA plate reader to calculate the percent of cell viability (Bio-Rad, Hercules, CA, USA). 

### 2.4. Determination of Cytotoxicity by Lactate Dehydrogenase (LDH) Release Assay

Cytotoxic effect of rutin in cervical cancer Caski cells was measured by lactate dehydrogenase activity using according to the manufacturer’s protocol (Cytotoxicity Cell Death Kit, Sigma, St. Louis, MO, USA). Briefly, 1 × 10^4^ cells/well Caski cells were seeded in 96-well culture plates and treated with different concentrations of rutin (60–180 µM) for 24 h. At the end of incubation, the supernatants were collected to assess the LDH activity and absorbance was recorded at 490 nm on a microplate reader (Bio-Rad, Hercules, CA, USA).

### 2.5. Determination of Morphological Changes by Phase-Contrast Microscopy

The effect of rutin against cervical cancer Caski cells was analyzed by observing morphological alteration. Caski cells have plated a density of 5×10^3^cells/well in 96-well plates and were incubated for 24 h. After this incubation, cells were exposed with various doses of rutin (60–180 µM) for 24 h and visible alterations in the cellular morphology of the cervical cancer cells were examined under inverted phase-contrast microscopy.

### 2.6. Estimation of Apoptosis via DAPI Staining

To analyze the effect of rutin on apoptosis in cervical cells, DAPI staining was used [24]. Caski cells were cultured (2 × 10^4^ cells per well) into 96-well plates and incubated at 37 °C for 24 h. Then, cells were treated with selected doses of rutin (90–150 µM) for about 24 h. Afterward, cells were fixed in ice-cold methanol for 10 min and subsequently permeabilized with permeabilizing buffer (3% paraformaldehyde and 0.25% Triton X-100). Finally, cells were stained with DAPI dye for 1 h and were observed under fluorescence microscope (EVOS FLoid Cell Imaging Station, ThermoFisher Scientific, Waltham, MA, USA). 

### 2.7. Mitochondrial Membrane Potential (MMP) Analysis

To assess the impact of rutin on mitochondrial membrane potential in cervical cells, Mito Tracker Red CMX Ros staining was employed [24]. Cervical cancer Caski cells were seeded (2 × 10^4^ cells per well) into 96-well plates and incubated at 37 °C for 24 h. After that, cells were exposed to different doses of rutin (90–150 µM) for about 24 h. Afterward, cells were permeabilized with permeabilizing buffer (3% paraformaldehyde and 0.25% Triton X-100), stained with Mito Tracker Red dye for 30 min, and observed under fluorescence microscope (EVOS FLoid Cell Imaging Station, ThermoFisher Scientific, Waltham, MA, USA).

### 2.8. Estimation of Caspase-9 and -3 Activities

To monitor the effects of rutin on caspase-mediated apoptosis, caspase-9 and -3 activities were determined by Colorimetric Assay Kits (BioVision, Milpitas, CA, USA). Briefly, treated and untreated cells (3 × 10^6^) were lysed in cell lysis buffer (ice-cold) for 10 min and then the cell lysate was centrifuged to collect the supernatant. Furthermore, 50 μL lysate was transferred into 96-well plates with 50 μL of reaction buffer, comprising 10 mM DTT. Afterward, 5 μL of the 4 mM substrate of each caspase was added in each well, incubated at 37 °C for 1 h, and the absorbance recorded at 405 nm on a microtiter plate reader. Finally, the % increase in caspase-3 and -9 activities was quantified by comparing the result with the level of the uninduced control.

### 2.9. Reactive Oxygen Species (ROS) Generation Analysis

In this experiment, cervical cancer Caski cells were seeded and treated with various doses of rutin (90–120 μM) for 12 h at 37 °C. Furthermore, cells were stained with 2′,7′-dichlorofluorescein diacetate (DCFH-DA) (25 μM) for 30 min in dark and observed under fluorescence microscope (EVOS FLoid Cell Imaging Station, ThermoFisher Scientific, Waltham, MA, USA). For quantitative estimation, cells (2 × 10^4^) were seeded in 96-well black bottom plates, exposed to rutin and stained with DCFH-DA (25 μM). Finally, fluorescence intensity was measured by using a multiwell microplate fluorimeter (Synergy Hybrid Multi-Mode Microplate Reader, BioTek, Winooski, VT, USA). 

### 2.10. Effect of NAC (N-acetyl-L-cysteine) on Cell Viability and ROS Generation

To investigate the ROS-mediated growth inhibitory effects of rutin NAC, a ROS inhibitor was used. As described in the previous sections, Caski cells were pre-treated with 10 mM NAC followed by rutin and stained with DCFH-DA for 30 min at 37 °C. Finally, fluorescence intensity was measured by using a multiwell microplate fluorimeter (Synergy Hybrid Multi-Mode Microplate Reader, BioTek, Winooski, VT, USA). To further examine the effect of ROS generation on cell growth inhibition in rutin-treated Caski cells, we performed an MTT assay in the presence of NAC (10 mM).

### 2.11. Cell Cycle Analysis

For evaluating the distribution of Caski cervical cancer cells in different stages ofthe cell cycle, flow cytometric analysis was carried out [25]. In brief, test cells were cultured in a 60 mm culture dish for 24 h and exposed for 24 h to various doses of rutin (90–150 µM). After incubation, cells were harvested, washed twice with PBS, and treated with 50 µg/mL solution of RNase A. Subsequently, cells were fixed with chilled 70% ethanol at −20°C for 1 h, washed again with cold PBS and stained with propidium iodide (PI) dye (25 µg/mL) followed by incubation at room temperature for 30 min. Finally, cell cycle distribution of cells was carried out by using a flow cytometer (Becton Dickinson FACSCalibur, San Jose, CA, USA). 

### 2.12. Real-Time qPCR Analysis

The RNA extraction of treated and untreated cells was performed using the HiPurATM Total RNA Miniprep Purification Kit (Himedia, Mumbai, India). The cDNA synthesis was performed using Verso cDNA synthesis kit (ThermoFisher Scientific, Waltham, MA, USA) and the mRNA expression level of target genes was estimatedby using DyNAmoColorFlash SYBR Green qPCR Kit (ThermoFisher Scientific, Waltham, MA, USA).The primer sequences target for this study were as follows:

*Bax* 5′-AAGAAGCTGAGCGAGTGT-3′   5′-GGAGGAAGTCCAATGTC-3′ (Accession number: NG_012191); *Bcl2* 5′-TCCATGTCTTTGGACAACCA; 5′-CTCCACCAGTGTTCCCATCT-3′ (Accession number: NM_000633.2); CyclinD1   5′-TGTGTGCAGAAGGAGGTCC-3′   5′-CCTTCATCTTAGAGGCCACG-3′ (Accession number: NM_012142.4); *CDK4*   5′-AGTGTACAAGGCCCGTGATC-3′   5′-ACGAACTGTGCTGATGGGAAG-3′ (Accession number: NM_000075.3); *Notch*-*1*   5’-GCAGTTGTGCTCCTGAAGAA-3’   5’-CGGGCGGCCAGAAAC-3’ (Accession number: NM_017617); *Hes*-*1* Forward Primer: 5′-ATTCTGGAAATGACAGTGAAGCAC-3′   Reverse Primer: 5′-CACCTCGGTATTAACGCCCTC-3′ (Accession number: NM_005524.3); and GAPDH   Forward Primer: 5’-GAAGGTCGGAGTCAACGGAT TTG GT-3’   Reverse Primer: 5’-CATGTGGGCCATGAGGTCCACCAC-3’ (Accession number: NM_002046.5). GAPDH (Glyceraldehyde-3-phosphate dehydrogenase) gene was used as an internal control and the data was evaluated by the comparative 2^−ΔΔCt^ threshold cycle. 

### 2.13. Statistical Analysis 

Experiments performed thrice with similar findings and represented by mean ± SEM (standard error mean). Statistical analysis was performed by one-way ANOVA using Dunnett’s multiple comparison post hoc test (* *p* < 0.01, ** *p* < 0.001 represent significant difference compared with the control).

## 3. Results

### 3.1. Antiproliferative and Cytotoxic Effects of Rutin against Cervical Cancer Caski Cells

**Figure 1:**
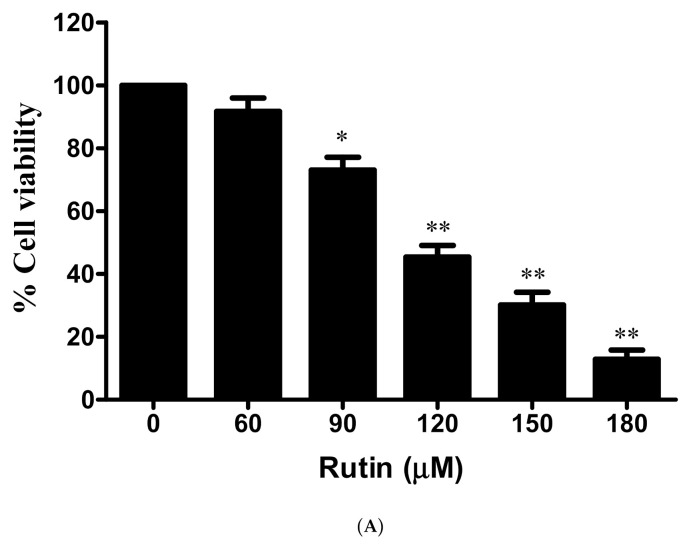

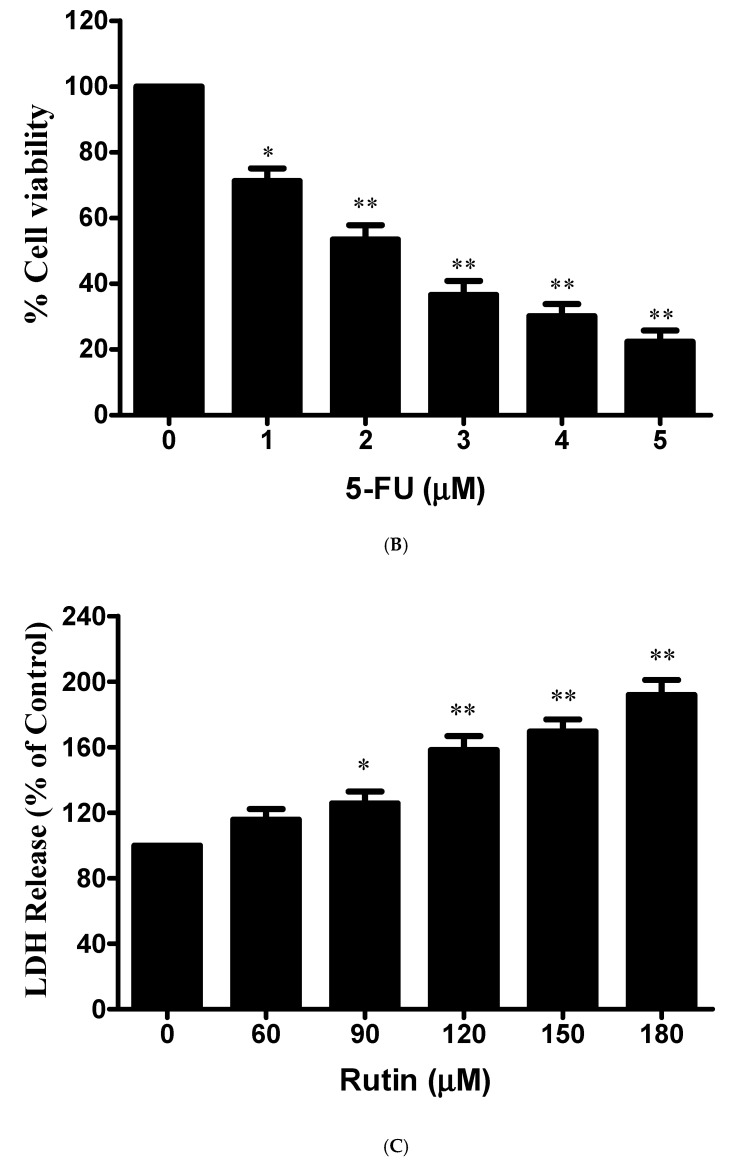

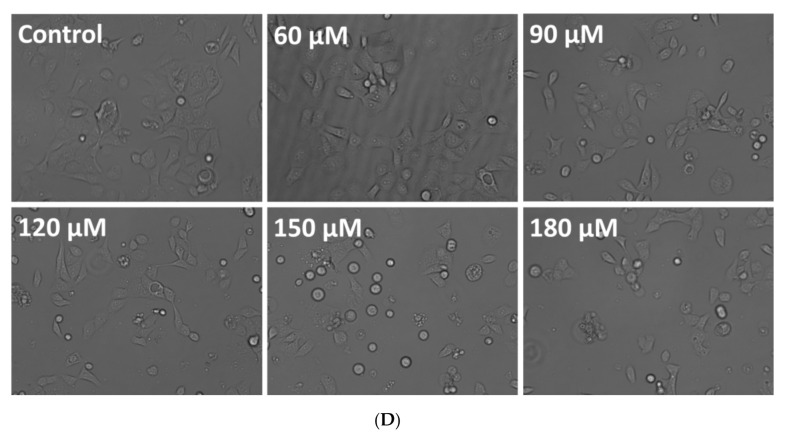
In vitro effect of rutin on cell viability in human cervical cancer Caski cells: (**A**) percent cell viability of Caski cells with rutin (0-180μM); and (**B**) 5-FU for 24 h and determined by MTT assay; (**C**) cytotoxic effects of rutin-treated Caski cells assessed by lactate dehydrogenase release assay; (**D**) morphological analysis of rutin-treated cells (0–180 μM) performed by phase-contrast microscopy. Experiments were performed thrice with similar findings and are represented by mean ± SEM (standard error mean). * (*p* < 0.01), ** (*p* < 0.001).

The antiproliferative effects of rutin on Caski cervical cancer cell was assessed by MTT cell viability assay. In Figure 1A, 24 h pre-treatment of rutin with increasing doses results in a significant reduction in the number of viable cells of Caski cells in a dose-dependent manner. Furthermore, cytotoxic effects of a standard drug 5-FU were also analyzed, and the results showed that 5-FU treatment resulted in a significant decrease in cell growth in a dose-dependent manner (Figure 1B). In addition to this, a cytotoxicity assay was carried out to confirm cell cytotoxicity by estimating the release of LDH via disrupted cell membrane. Results revealed a significant increase in dead cells after the treatment of rutin for 24 h (Figure 1C). Thus, these findings of the cell proliferation and cytotoxicity assay strongly confirm the anticancer potential of rutin against cervical cancer Caski cells. 

### 3.2. Effect of Rutin on Caski Cell Morphology 

To further observe the morphological deviations in rutin-treated Caski cells, inverted phase-contrast microscopy was performed. Photomicrographs demonstrated that rutin treatment resulted in cell shrinkage and detachment from the surface in a dose-dependent manner in comparison to the normal morphology of healthy Caski controls (Figure 1D).

### 3.3. Rutin-Induced Apoptosis in Caski Cervical Cancer Cells 

**Figure 2:**
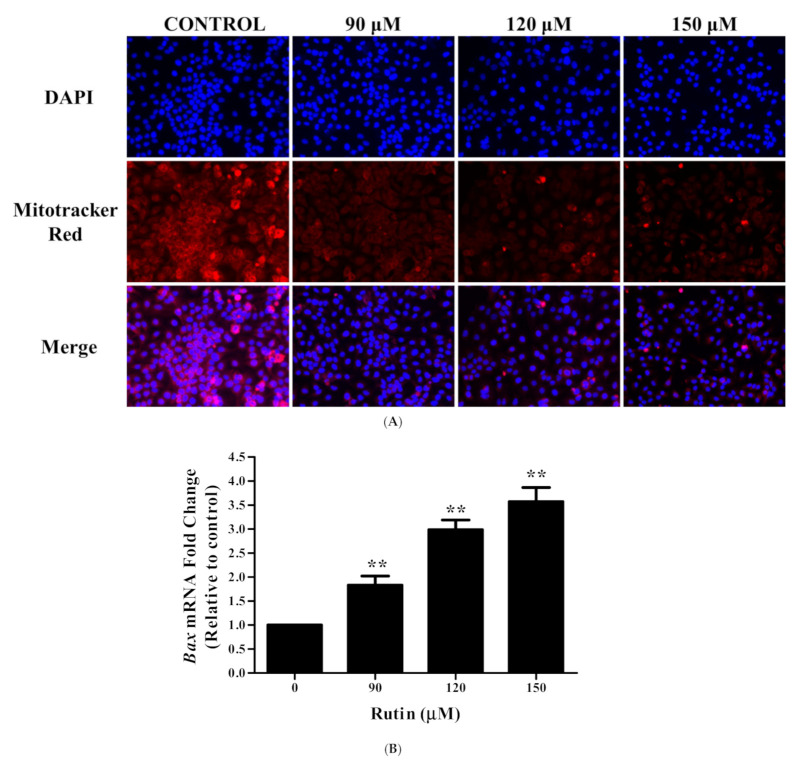

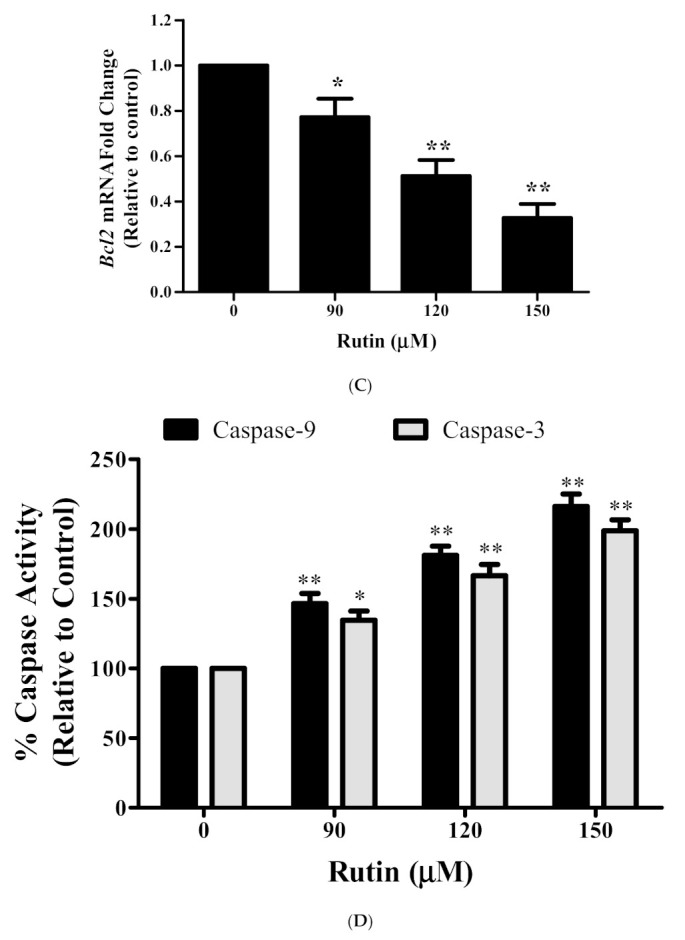
Effects of rutin on apoptotic cell death in human cervical cancer Caski cells: (**A**) analysis of nuclear morphology and mitochondrial membrane potential of cervical cancer Caski cells evaluated by fluorescence microscopy using DAPI and Mitotracker Red staining dye, respectively; (**B**) effect of rutin (0–150 μM) on the mRNA expression of apoptosis-related genes Bax; and (**C**) Bcl2 assessed by quantitative real-time PCR; (**D**) percent caspase-3/-9 activity in cervical cancer Caski cells after rutin treatment (0–150 μM) for 24 h evaluated by colorimetric method. Experiments were performed thrice with similar findings and are represented by mean ± SEM (standard error mean). * (*p* < 0.01), ** (*p* < 0.001).

To establish whether cell proliferation was inhibited by the apoptosis effects—induced by rutin—DAPI staining was carried out. The formation of apoptotic bodies and nuclear condensation was revealed by DAPI staining in rutin-treated Caski cells (Figure 2A). Moreover, the photomicrographs showed that the apoptosis-inducing potential of rutin was dose-dependent, as the permeability of cells undergoing apoptosis was found to be significantly increased with higher rutin doses.

### 3.4. Rutin Induces Mitochondrial Membrane Depolarization in Caski Cells

To further reveal the underlying apoptosis pathway, the MMP was determined in rutin-treated Caski cells by the Mitotracker red stain. Figure 2A exhibited that there was a substantial decrease in MMP in Caski cells, which was evidenced by red fluorescence reduction in a dose-dependent manner. These results suggested that mitochondrial membrane depolarization could be one of the mechanisms behind apoptotic induction in rutin-treated Caski cells. 

### 3.5. Rutin Modulated the mRNA Expression Apoptotic-Related Genes 

To achieve additional insight into the mechanism of apoptosis induced by rutin, we assessed the mRNA expression of apoptosis-related genes in Caski cells after rutin treatment. Results demonstrated that the level of pro-apoptotic gene *Bax* increased significantly in rutin-treated Caski cells line compared with untreated control cells. Furthermore, the level of anti-apoptotic gene *Bcl2* revealed a considerable reduction in rutin-treated Caski cells (Figure 2B,C).

### 3.6. Rutin Activates Caspase-3/-9 in Caski Cells

The quantification of caspase activity is considered an important aspect of apoptosis. To confirm the potential of rutin in caspase-mediated apoptotic induction, the caspase-3/-9 colorimetric assay kit was used. Caski cells were treated with different doses of rutin and analyzed for the induction of caspase-3 and 9 enzymes. Figure 2D showed that rutin markedly increased the caspase-3 and caspase-9 activity in cervical cancer Caski cells.

### 3.7. Augmented ROS Generation by Rutin Contributes to Apoptosis 

**Figure 3:**
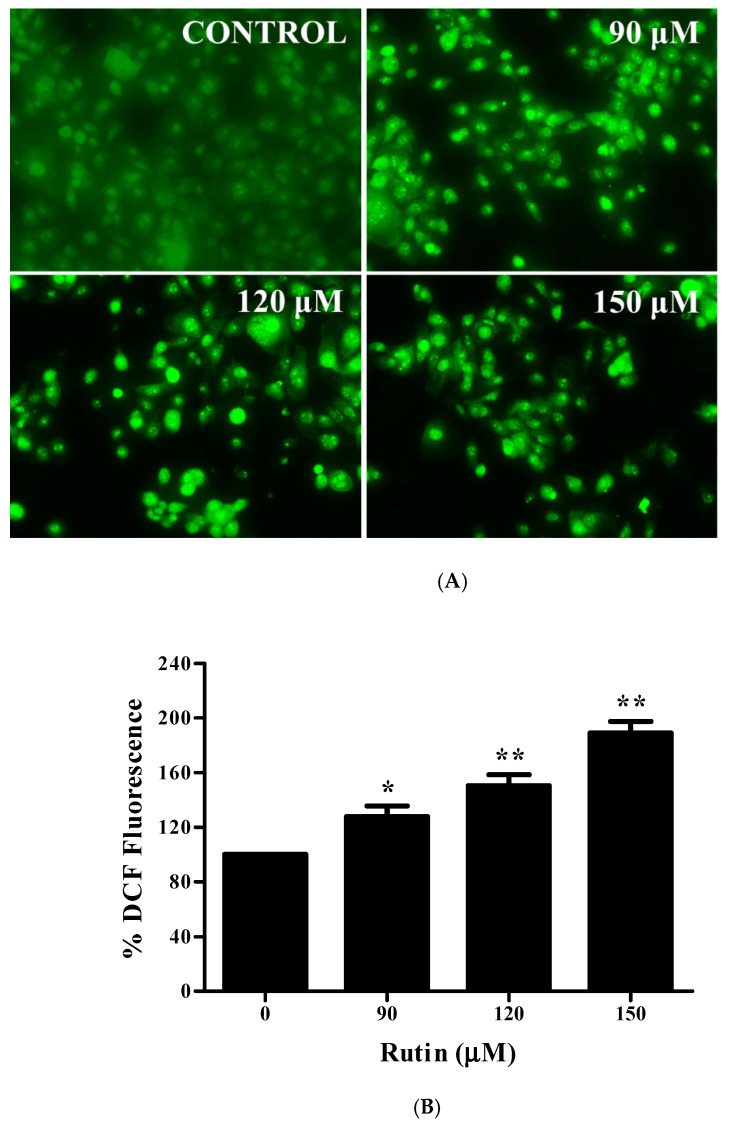

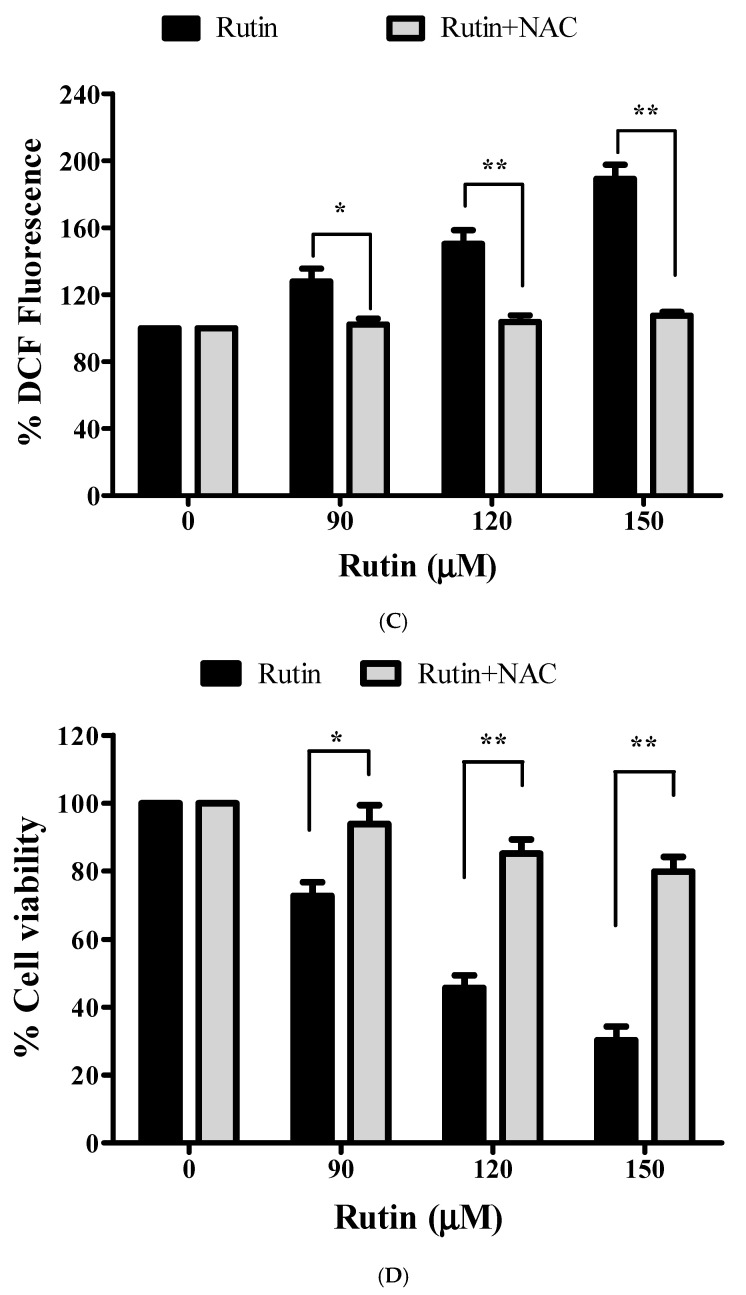
Effect of rutin on ROS production in human cervical cancer Caski cells: (**A**) augmented intracellular ROS generation in rutin-treated cervical cancer Caski cells were evaluated by fluorescence microscopy using DCFH-DA staining; (**B**) ROS quantification in terms of percent fluorescent intensity in rutin-treated cervical cancer Caski cells; (**C**) ROS scavenging in rutin-treated cervical cancer Caski cells pre-treated with 10 mM NAC; (**D**) restoration of cell viability in rutin-treated cervical cancer Caski cells pre-treated with 10 mM NAC assessed by MTT assay. Experiments were performed thrice with similar findings and are represented by mean ± SEM (standard error mean). * (*p* < 0.01), ** (*p* < 0.001).

Mitochondrial dysfunction can be initiated by many factors including ROS generation, which may result in apoptotic induction [26]. Subsequently, the effect of rutin on intracellular ROS stimulation was analyzed by using DCFH-DA fluorescent probe in Caski cancer cells after 12 h of treatment. Rutin treatment has led to an increased level of ROS generation in Caski cells as compared to the control (Figure 3A). The results of quantitative investigation also revealed that 90 μM of rutin augments the ROS generation, i.e., about 27.88% with concerning the control. Furthermore, 120 and 150 μM treatment of rutin resulted in ROS generation to approximately 50.35 and 89.07%, respectively (Figure 3B). Furthermore, to elucidate the role of rutin-mediated ROS generation in apoptosis induction, the quantification of the ROS level was carried out in cervical cancer cells, pre-treated with NAC. The results revealed an approximately complete reduction in ROS generation by pre-treatment with NAC (10 mM) (Figure 3C). These findings suggested that rutin induces apoptotic cell death by an oxidative stress-mediated pathway.

### 3.8. Restoration of Cell Viability by N-acetyl-L-cysteine (NAC)

To examine the contribution of ROS in rutin-induced cell growth reduction, Caski cells were exposed to various concentrations of rutin in the absence/presence of NAC (10 mM). Pre-treatment with NAC significantly restored the number of viable cells caused by rutin, as evidenced in Figure 3D. After 24 h treatment with 90, 120 and 150 µM of rutin, NAC was capable of restoring cell viability from 72.7%, 45.69 % and 30.28% to 93.85 %, 85.14 %, and 79.87%, respectively (Figure 3D). Thus, these results suggested the association of ROS generation with rutin-induced apoptosis and decreased cell proliferation.

### 3.9. Rutin Treatment Induces G0/G1 Arrest in Caski Cells

**Figure 4:**
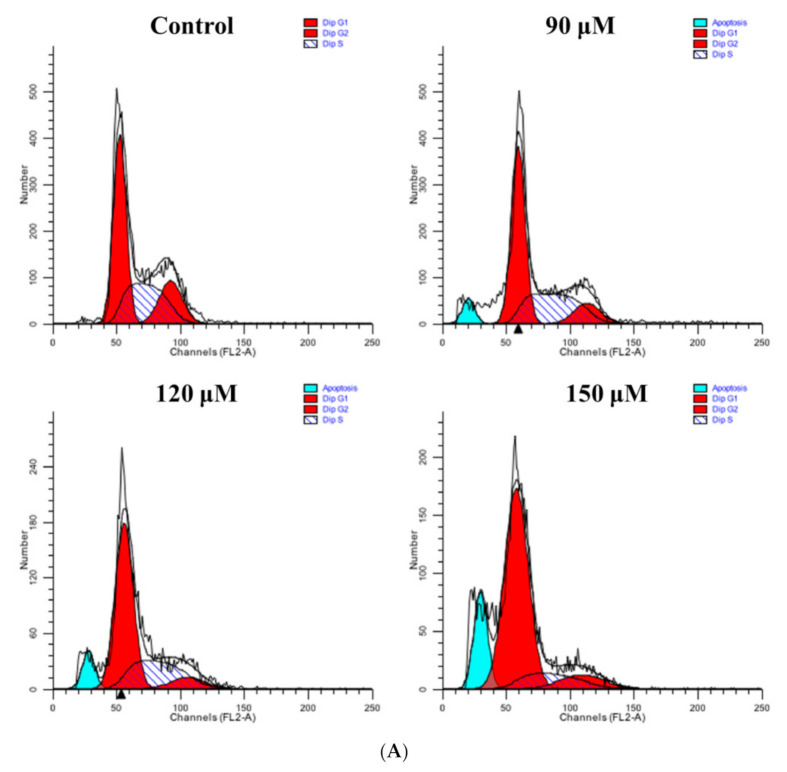

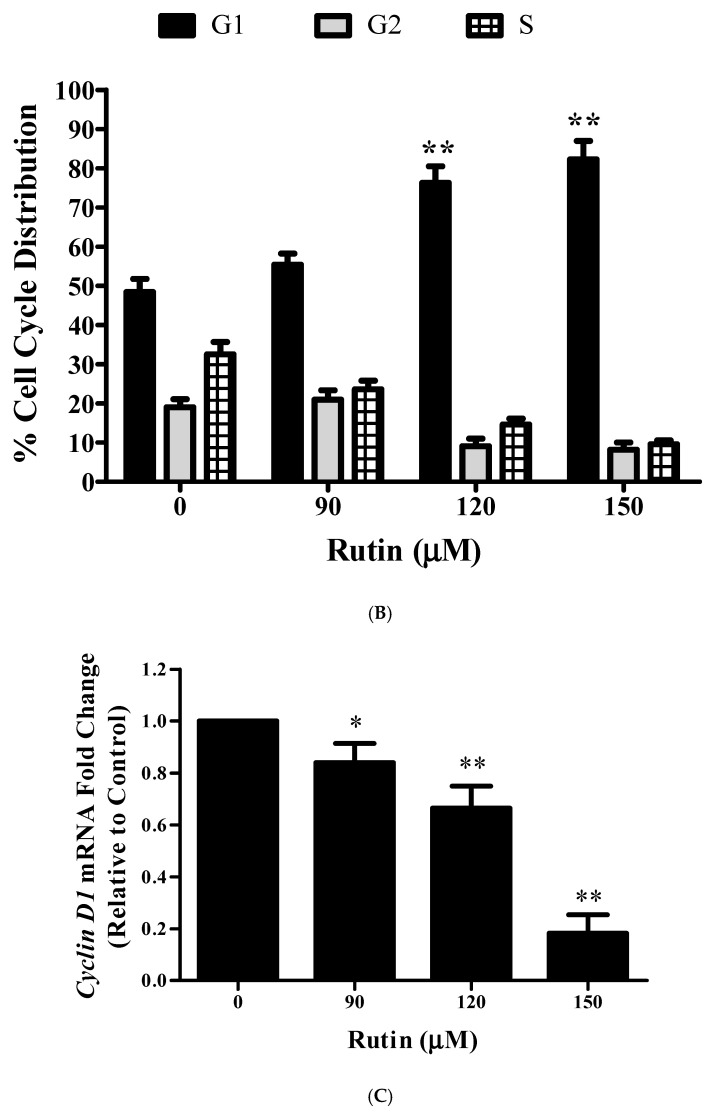

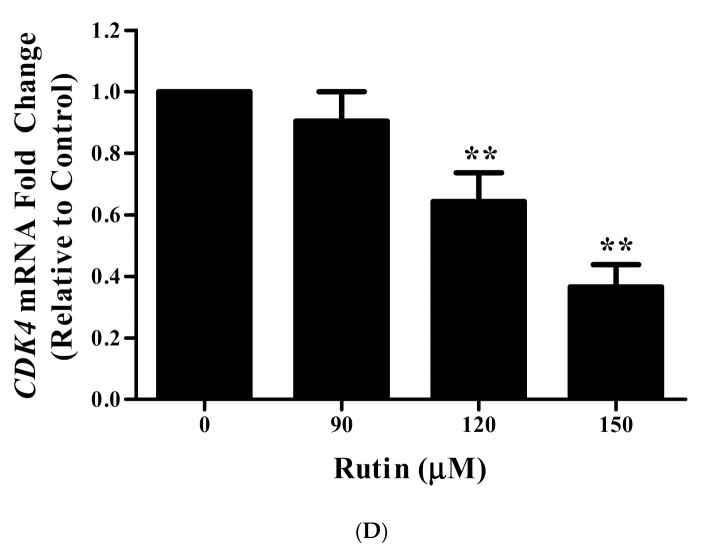
Effect of rutin on cell cycle modulation in human cervical cancer Caski cells: (**A**) histogram showing the cell cycle percentage of Caski cells in each phase; (**B**) percent cell cycle phase distribution of rutin-treated Caski cells and growth arrest at G0/G1 phase; (**C**) effect of rutin (0–150 μM) on mRNA expression of cell cycle-related genes Cyclin D1; and (**D**) CDK4 assessed by quantitative real-time PCR. Experiments were performed thrice with similar findings and are represented by mean ± SEM (standard error mean). * (*p* < 0.01), ** (*p* < 0.001).

The cell growth inhibition could be due to the cell cycle arrest or apoptotic induction in cancer cells [27]. To monitor the effects of rutin on changes in cell cycle distribution, flow cytometry examination was conducted with PI dye (containing RNase). The results revealed that the G0/G1 fraction of cells was significantly increased in a dose-dependent manner after treatment with varying concentrations of rutin in Caski cells (Figure 4A). The percentage of Caski cells in the G0/G1 phase of cell cycle were found to be 55.43%, 76.29%, and 82.24% at the doses of 90, 120, and 150 μM of rutin, respectively (Figure 4A,B).

### 3.10. Rutin Regulates CyclinD1 and CDK4 mRNA Expression

To further enlighten the regulatory mechanism of rutin on the cell cycle, we investigated the changes in the mRNA expression level of *Cyclin D1* and *CDK4* (cyclin-dependent kinase 4) following 24 h rutin treatment by qRT-PCR analysis (Figure 4C,D). The results indicated that rutin decreased the mRNA expression of *Cyclin D1* and *CDK4*, which suggested that rutin could halt the progression of the cell cycle at G0/G1 phase through the regulation of *CDK4* and CyclinD1 gene expression. 

### 3.11. Rutin Treatment Downregulated mRNA Expression of Notch-1 and Hes-1 in Caski Cells 

**Figure 5:**
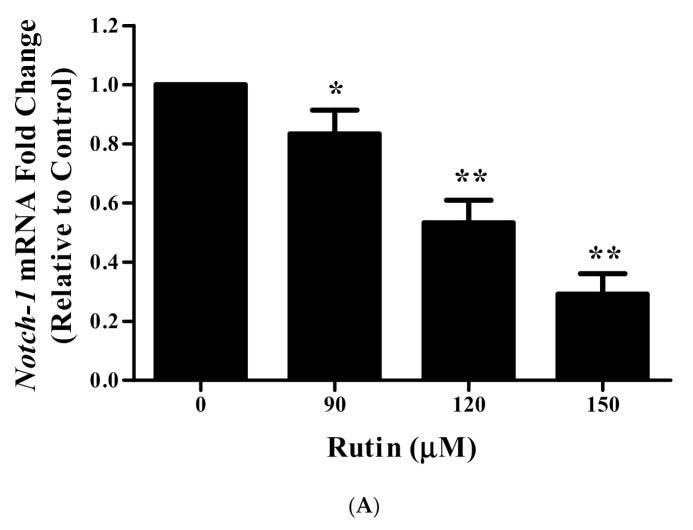

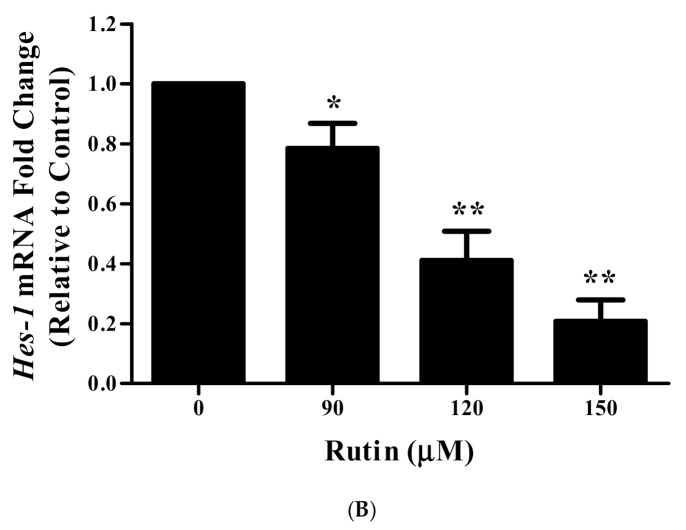
Effect of rutin on the modulation of two key targets (Notch-1 and Hes-1) of the Notch signaling pathway in human cervical cancer Caski cells: (**A**) decreased mRNA expression of the Notch signaling receptor Notch-1 in rutin-treated Caski cells; (**B**) decreased mRNA expression of the Notch signaling downstream transcription factor Hes-1 in rutin-treated Caski cells estimated by using quantitative real-time PCR. Experiments were performed thrice with similar findings and are represented by mean ± SEM (standard error mean). * (*p* < 0.01), ** (*p* < 0.001).

To provide insight into the association of the apoptosis-inducing effects of rutin with two key targets, *Notch*-*1* and *Hes*-*1*, we examined the mRNA expression by qRT-PCR. Upon the treatment of various doses of rutin with cervical cancer Caski cells, a significant reduction in the mRNA expression of both genes was observed (Figure 5A,B).

## 4. Discussion

In recent years, there has been increasing interest in exploring the anticancerous potential of natural compounds against several types of carcinomas. Among them, plant-derived bioactive compounds have revealed remarkable effects in both preclinical and clinical [28,29]. The scientific community has currently recognized plant’s flavonoids to be a distinctive group of therapeutic substances due to their various pharmacological properties [30,31,32]. Amongst them, rutin has presented diverse health benefits, such as, antimicrobial, antioxidant, anticancer, anti-diabetic activities, and cardiovascular protective properties [33,34,35,36]. Herein, we explored the mechanism of rutin-induced apoptosis by modulating the key molecules of a Notch signaling pathway in human cervical cancer Caski cells. 

In preliminary studies, the antiproliferative effects of rutin were confirmed by MTT and LDH release assays, and the results showed that this natural compound resulted in a significant reduction in viable cells as well as significant alterations in cellular morphological structure confirming the previous report [20,37]. Additionally, standard drug 5-FU treatment also resulted in significant cell growth inhibition but with more cytotoxic effects on normal cells in comparison to growth inhibitory potential of rutin (Figure 1A–D) [20,38]. Altogether, these findings supported our concept that rutin would probably emerge as a safer drug candidate against human cervical cancer cells.

The induction of apoptosis may result in DNA damage, the cleavage of PARP, and the loss of mitochondrial membrane potential which leads to aberrant cell growth and stimulates the risk of cancer development [39,40]. DAPI staining further suggested that rutin induced a significant nuclear condensation in Caski cells (Figure 2A). Chemotherapeutic agents trigger an intrinsic pathway of apoptosis that depends on pore-forming, mitochondrial membrane potential, pro-apoptotic *Bcl2* family proteins (*Bax* and Bak), and caspase activation [41,42]. Hence, we analyzed the early events of apoptosis by determining the membrane potential of mitochondria, where our results revealed the depolarization of MMP by reducing the fluorescence intensity in a dose-dependent manner (Figure 2A). Then, to gain more insight into the mechanism of apoptosis, we explored the expression of apoptosis-related genes. The mitochondrial pathway (intrinsic) of apoptosis is accomplished by the release of cytochrome C from mitochondria, in which anti-apoptotic *Bcl2* and pro-apoptotic *Bax* proteins play a crucial role [43]. Our results on Caski cells displayed that owing to rutin treatment, the mRNA expression of the *Bax* gene was significantly upregulated, and simultaneously, the expression of *Bcl2* gene was downregulated, which favored the disruption of MMP, leading to apoptosis induction (Figure 2B, C). Increasing evidence has suggested a direct association between the loss of MMP and ROS generation, consequently resulting in the apoptotic induction in cancer cells [44,45]. Our results showed that rutin-induced ROS production is an important aspect in its antitumor and apoptosis-inducing effect. Additionally, the rutin-mediated enhanced intracellular ROS generation in cervical cancer cells was reduced by an ROS inhibitor (NAC) as well as the restoration of cell viability, which further confirmed the generation of ROS due to glycyrrhizin treatment (Figure 3A–C). Moreover, it has also been a fact that ROS-induced MMP depolarization results in the release of mitochondrial contents and caspase activation, which are ultimately responsible for apoptotic induction [46]. The present study also revealed that rutin treatment in cervical cancer Caski cells stimulates caspase activity, as evidenced by the increased activation of caspase-3 and -9, and especially the caspase-3 activity compared to the control after 24 h (Figure 2D). Overall, these results exhibited that rutin-induced apoptosis in the Caski cells is mitochondrial (intrinsic pathways) mediated.

The cell cycle is characterized by a cascade of regulated events needed for cell division and DNA replication which cycles between quiescence and cell proliferation phases (G0↔M phases) [47,48,49]. Dysregulated cell cycle progression is the crucial aspect leading to the uncontrolled cell proliferation and development of cancers [50]. Our flow cytometric results for cell cycle phase distribution in rutin-treated Caski cells exhibited significant cell cycle arrest at the G0/G1 phase (Figure 4A,B). Several studies suggested that cell cycle progression is directly controlled by the cyclins and CDK family proteins [51]. It is a well-established fact that the modulated expression of cell cycle-related genes can induce uncontrolled proliferation in cancer cells. The results revealed that treatment with rutin significantly downregulated *Cyclin D1* and *CDK4* mRNA expression, which suggested that rutin can stimulate the cell cycle arrest (G0/G1 phase) (Figure 4C,D).

**Figure 6:**
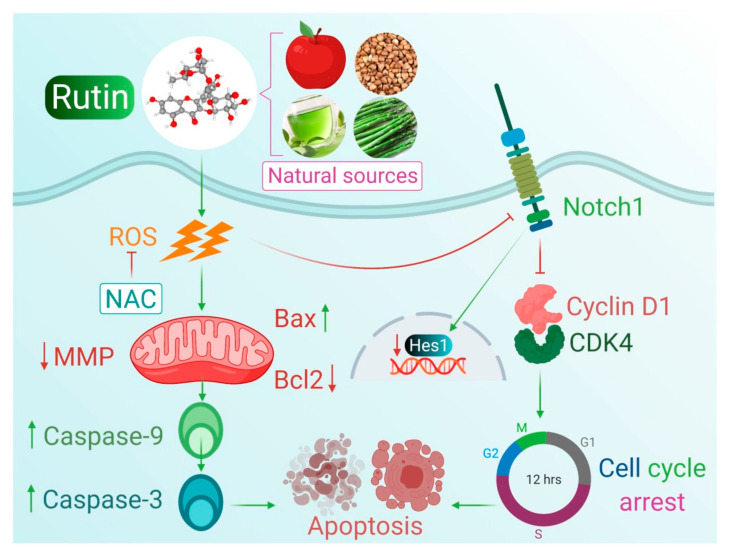
Proposed mechanisms of in vitro apoptosis-inducing effects of rutin on cervical cancer Caski cells.

Cell signal transduction consists of a vast array of signaling molecules (molecular signals and exogenous stimuli) that can modulate intracellular signaling via changes in target genes’ expression inside the nucleus. Normally, these signal transduction pathways regulate cellular differentiation, growth, and apoptosis but the abnormal activation of these signal pathways resulted in a cascade of abnormal biological modifications which ultimately leads to the malignancy of cells [52,53,54]. Recent research focused on the molecular biology of cancer cells by targeting important targets of the signaling pathway. Numerous reports have suggested that the dysfunction of the Notch signaling pathway is frequently associated with the occurrence and progression of multiple types of carcinomas [55,56,57]. The abnormal activation of Notch signal transduction is closely linked with the suppression of apoptosis by *Bcl2* protein activation as well as with the unusual activation of other pathways [58,59]. Previous reports also suggested that the aberrant activation of this signal transduction resulted in increased cell cycle progression through the modulation of expression of cell cycle proteins including *CDK*-*2*, *CDK* inhibitors, *Cyclin D1*, and SKP2 proteins [59,60,61,62]. Our previous studies also reported the apoptosis-inducing efficacies of natural products, and their association with the modulated expression level of several key molecules (such as *Notch*-*1*, *Jagged*-*1* and *Hes*-*1)* of Notch signaling pathway [63,64]. These reports further motivated us to explore the apoptosis-inducing potential of rutin via targeting two major molecular targets of Notch signaling pathways including *Notch*-*1* and *Hes*-*1* in Caski cells following our previous findings presenting rutin efficacy in the cervical cancer HeLa cell line [65]. Results indicated that rutin treatment significantly downregulated the mRNA expression of *Notch*-*1* and *Hes*-*1* genes of Notch signal transduction in human cervical cancer Caski cells (Figure 5A,B). In conclusion, it can be stated that rutin downregulated them RNA expression level of two crucial targets of the Notch signaling pathway, and thereby prompted apoptosis to halt the growth and viability of cervical cancer Caski cells (Figure 6). 

## 5. Conclusions

Overall, the present findings suggest that rutin downregulated the expression of *Notch*-*1* and *Hes*-*1* and induce apoptosis via modulating apoptotic-related genes (*Bax*, *Bcl2*) and cell cycle-related genes (*Cyclin D1* and *CDK4*) which would ultimately arrest cell cycle progression. Altogether, rutin could be considered as a potent therapeutic agent for the management of cervical cancer; however, further investigations into its anticancer properties in animal models and clinical exploration are still required.

## Data Availability

The data supporting the results of this study are available on reasonable request.
